# Beyond “Error-Correction”

**DOI:** 10.3389/fpsyg.2012.00423

**Published:** 2012-10-17

**Authors:** Paco Calvo, John Symons, Emma Martín

**Affiliations:** ^1^Departamento de Filosofía, Universidad de MurciaMurcia, Spain; ^2^Department of Philosophy, University of KansasLawrence, KS, USA; ^3^Department of Philosophy, University of ConnecticutStorrs, CT, USA

Building upon Ashby’s claim that “The whole function of the brain is summed up in: error-correction,” Clark elaborates an integrative “hierarchical generative model” of the mind/brain. A Helmholtzian worldview permeates Clark’s model insofar as it presupposes an inherently ambiguous relationship between mind and world; a relation that calls for an inferential treatment of perception and action. In this short paper, we note that embodied cognition offers a wider range of approaches to cognition and perception than error-correction alone. Two areas of research – the synthetic approach to embodied cognition (Pfeifer et al., 2008) and direct learning (Jacobs and Michaels, 2007) – serve to highlight what exclusive attention to error-correction misses. Our view is that perception and action are not reducible to error-correction.

Consider the role of the structure of the body in action. Clark assumes that action operates in a manner similar to perception with the exception that the reduction of error obtains in part non-neurally by moving around and thereby altering the shape of proprioception and sensory input. But actions involve more than action-oriented predictive processing. The synthetic approach to embodied cognition explores the role of physical structure itself. An example is the “Yokoi hand,” a prosthetic hand designed with deformable materials, such as flexible and soft gripping surfaces or artificial tendons arranged in a particular morphological structure (Yokoi et al., 2004). A single instruction from the hand’s control system can initiate a range of kinds of gripping actions with a range of different kinds of objects. In this case, the morphological design itself secures that finger tips come together as the hand gets closed. Likewise, the very material composition of the fingertips conforms to the shape of the object to be gripped. The morphology of the Yokoi hand carries the burden of coping with a wide range of behavioral challenges, and the need for top-down control, or functions based on expectations, for that matter, reduces dramatically.

It may be argued nonetheless that this approach only works for organisms with a relatively limited behavioral repertoire; a setting in which it is easier to imagine all evolutionarily relevant computational challenges being resolved via the physical structure of the body itself. For agents with more complicated sets of behaviors and challenges, it is reasonable to assume the involvement of some kind of error-driven control system that allows for learning to take place. Nevertheless, explanations of action will remain incomplete if they do not account for the kinds of morphological problem solving undertaken by organisms at a variety of levels.

More significantly for the advocate of error-correction are forms of direct learning (Jacobs and Michaels, 2007); a natural extension of direct perception that developed from the appreciation that perception fails to account inferentially for the adaptive capability of organisms (Jacobs and Michaels, 2002). Strikingly, direct learning can do away with the need to minimize errors altogether. How is this possible? The error-correction view appears to be unavoidable once one assumes that the world does not furnish agents with an answer to their needs. On the error-correction view, evolution equipped us with hierarchical generative neural models precisely for the purpose of resolving ambiguity. But research on direct learning, inspired by ecological psychology (Gibson, 1979), and in particular by the theory of direct perception, does not bite the Helmholtzian bullet of an inherent ambiguous mind-world linkage. Instead, constraints that operate at an ecological scale between organism and environment allow for information to be *specificational*. Tau theory (Lee, 1976) with the direct pickup of informational invariants in ambient energy flows, provides the canonical illustration of a theory that takes this tack. The ecological task then is to discover the type of information that is specificational for the non-inferential resolution of the perceptual problem in question.

Clark’s essay does not contain a single reference to Gibson’s work. It is possible that Clark endorses the common belief that Gibsonian principles might work for perception (best-case scenario), but that they are not operant when it comes to learning. After all, his is a theory of cognition proper, not of perception. In this way, regardless of our success in discovering specificational information, we are not able to *learn* non-inferentially. Rather, we learn because we are able to correct our brain’s guesses.

How can we possibly learn, if learning entails some form of experience-driven change in the behavior of an organism, and the perception of affordances is direct?

According to direct learning, learning-driven changes are *themselves* specific to Gibsonian environmental properties. Note however that for any model to constitute a genuine cognitive architecture, with forethought as a hallmark, perception and action are to be integrated somehow. It is reasonable to do so by honoring among other things the functional role of intention and attention, as Clark does in his hierarchical model. Interestingly, direct learning incorporates the functional role played by intention and attention in early perception and action. First, the intention of an organism, seen from the general ecological framework, sets the goals and boundary constraints of actions, and hence defines which actions can be considered well adapted. Intentions are, among other things, related to the needs of the organism. Second, attention, understood ecologically, refers to the informational basis of perception and action at a particular moment. With respect to the adaptation of the organism to the environment, the *education of intention* is defined as the process by which organisms improve the goals that they set for their actions, and the *education of attention* can be understood to be the process by which organisms come to rely on patterns of information that, given the intention, can more adequately serve pragmatic purposes (Jacobs et al., 2012).

Clark endorses Marr’s (1982) view of the appropriate analysis of levels in cognitive systems. He describes his aim as to furnish cognitive science with “a systematic approach that addresses the levels of … the computation, the algorithm, and the implementation.” Overall, our complaint is that Clark’s model must assume ambiguity at the computational level, and representations at the algorithmic level. We hint at an alternative: the synthetic approach to embodied cognition and the neo-Gibsonian theory of direct learning are not committed to the computational-algorithmic levels by rule-following the approximation of a pre-established input-output function. In our view, the error of the “error-correction” approach resides in a Helmholtzian–Marrian failure to appreciate this point.
